# Analysis of partnerships between agricultural cooperatives and development actors: A national survey in Saudi Arabia

**DOI:** 10.1371/journal.pone.0270574

**Published:** 2022-06-24

**Authors:** Bader Alhafi Alotaibi, Hazem S. Kassem

**Affiliations:** Department of Agricultural Extension and Rural Society, King Saud University, Riyadh, Saudi Arabia; University of Salento, ITALY

## Abstract

The partnerships between agricultural cooperatives and development actors play a critical role in meeting development challenges and building cooperative sustainability. The objective of this study was to analyze the key characteristics of engagements established between agricultural cooperatives and other actors and determine their success level. An analytical framework was developed to highlight nine areas, namely partnership configuration, stakeholders, objectives of the partnerships, partnership types, partnership stages, communication methods, achieved outcomes, partnership evaluation, and partnership sustainability. The targets were all agricultural cooperatives building associations with other actors between 2016 and 2020 in Saudi Arabia. Therefore, the study covered 69 partnerships founded by 32 agricultural cooperatives. The results revealed that the cooperatives involved in partnerships essentially provide farming inputs and equipment for their stakeholders and capacity building and training purposes. The public sector was the leading actor that collaborated with agricultural cooperatives in inter-sector partnerships. The findings also showed that 55.1% of the partnerships were “strategic partnerships” in cases of both independent value formation and integrative partnerships. By focusing on mapping the partnerships, this study presents beneficial information for policy-makers working on how agricultural cooperatives dealt with the other actors and the lessons gathered to build future sustainability collaborations.

## 1. Introduction

The sustainability of nonprofit and for-profit organizations has acquired special attention in the literature over the last few decades [1–5]. United Nations agenda for sustainable development goals (SDGs) presents a framework comprising 17 SDGs for various organizations to tackle sustainability issues in social, economic, and environmental challenges [6, 7]. Overcoming these challenges demands constant improvement of capabilities, refined management of resources and assets, and a strong collaboration between actors [8, 9]. Partnerships (SDG 17–partnerships for the goals) pose a crucial opportunity to alleviate these needs by designing a platform for multi-stakeholders to address challenging issues collectively [10–12].

In the agricultural sector, a consensus is accepted among national and international partners that agricultural cooperation substantially contributes to achieving SDGs [13, 14]. This role may be noticed in efforts in poverty reduction and gender equality, access to quality education and life-long learning opportunities, financing and delivering healthcare services, improving food security, easing access to clean water and sanitation services, and sustainable management of natural resources [15–17]. Acquiring those goals needs a transformation toward new institutional arrangements through a coordinated effort by all stakeholders, such as agricultural cooperatives, nonprofit organizations, the private sectors, the government, and the international partners [18, 19]. These partnerships are effective strategies for forming a more sustainable and inclusive performance of agricultural cooperatives [20].

The research on partnerships increased substantially in the late 1990s thanks to governance structures addressing sustainable agricultural challenges, linking farmers to markets, and fostering capacity building [21, 22]. Partnerships are beyond collaborative arrangements among actors in the same sector or between sectors [23]. It aims to achieve independent outcomes for pooled resources and shared risks and responsibilities to produce added value [24, 25]. Thus, Austin and Seitanidi [26] underlined four values acquired due to a collaborative work: associational value; relating to benefit driven by a partner involved in a partnership with a specific actor, transferred resource value, indicating the outcomes gained by the partner due to receiving a resource from the other partner, interaction value; the indirect and intangible results coming from the collaboration, and lastly synergistic value; centering on comparing the results obtained from working collectively by combining resources to working separately. Hence, building sustainable partnerships demands a careful analysis of all the features affecting value optimization [20]. The partnership’s features included several elements such as reasons for partnering, objectives of partnering, types of the partners, the incentives for each party, the timeframe of the partnership, governance structure, and outcomes obtained. These elements were interrelated and varied depending on the partners involved and the specific context [27].

Even though the literature had many studies investigating the partnerships between nonprofit organizations and businesses from different aspects, little empirical research tackled the issue of how agricultural cooperatives or other forms of farmers’ groups collaborated with other actors in partnerships. It affects sustainability and results on the society and the cooperatives in the short and long term. Some of those studies underlined the strategies for supporting agricultural cooperatives to deal with other actors and factors affecting cooperatives’ participation [28–32]. However, the rest of them centered on a specific case study of partnerships to build agricultural value chains [19, 33–36] or partnerships for joint agricultural research, innovation, and technology transfer [37, 38], or partnerships for delivering business development services to agricultural cooperatives [39]. To our understanding, no studies explicitly supplied a holistic framework for analyzing the specific features of the partnerships built between agricultural cooperatives and other actors at the national level. Moreover, no studies have addressed this topic under the Saudi Arabia context. Therefore, the aim of the present study was to analyze the partnerships built between agricultural cooperatives and other actors at the national level from 2016 to 2020. The objectives were to specifying the characteristics of agricultural cooperatives–other actors’ partnerships, and exploring the outcomes of these partnerships were conducted.

## 2. Literature review

### 2.1. Agricultural cooperatives in Saudi Arabia

Saudi cooperative societies contain eight essential categories: multi-purpose, housing, agricultural, marketing, fishermen, services, vocational, and consumer [17]. The number of cooperative societies in 2020 was 245 cooperatives, 63 of which were agricultural cooperatives covering about 25% of the total cooperatives in Saudi society [40]. The cooperative sector formed approximately 1% of the Gross Domestic Product (GDP) [41]. In 2016, Saudi Arabia presented the 2030 vision, highlighting special attention to empowering the cooperative sector, aiming to increase its share in its GDP contribution to 5% by 2030 [42]. One of the strategic objectives given in the 2030 vision was to establish and reinforce the partnerships between cooperative societies and other actors [42]. Specifically, from 2016 to 2020, cooperatives used this opportunity. They increased the number of partnerships with public and private sectors to foster their growth and performance and respond to complex problems or sustainability issues [43].

At the end of 2019, the Ministry of Human Resources and Social Development reviewed and introduced many organizational and legal factors, human and administrative capabilities and started a new program for the cooperative sector, “Development of Cooperative Societies” [44]. This program was devised after a longitudinal study of the cooperative sector. It relied on the most prominent challenges it confronted and investigated the globally implemented standards to have the best practices aligning with the culture of the Kingdom of Saudi Arabia. This program aimed to boost the cooperative sector in four essential areas [44]: 1) developing an effective e-registration system in collaboration with the supervisory and the competent security authorities to decrease the registration period to a maximum of 60 days; 2) finding possible solutions with the relevant authorities to address the issue of the dual licensing system (cooperative—commercial) to handle cooperatives like small and medium enterprises possessing only one license (commercial license); 3) supplying innovative financing models (indirect financing) and supporting packages including tax exemptions and specific incentives as per the cooperative’s performance and its effectiveness in attaining specific goals set in advance, and lastly 4) forming new standards for partnerships to help partners construct explicit business models delivering greater chances for the cooperative’s success and growth.

### 2.2. Building partnerships for cooperative sustainability

The complicated nature of sustainability problems, the allocation of responsibilities and resources to different partners, and the inability of a single actor to overcome the developmental challenges suggest opportunities and challenges to agricultural cooperatives in all countries [45, 46]. The transformations are urgently needed for long-term sustainable systems and to allow the conditions for the partnership to occur [47]. According to Horan [48], the specific features of the partnerships in transformation processes had the potential to engage various stakeholders effectively and apply an integrated approach to collaboration. These transformations were essential parts of the broader social, economic, political, and organizational contexts encouraging the formation of a partnership [49]. Moreover, a critical role of information communication technologies in transforming a cooperative in the business environment was among the driving forces enabling cooperatives to partner [23]. However, challenges in the agrifood system–precisely the competitive market condition, modifying customers’ demand patterns, boosting interest in food safety and quality, the relevance of complying with standards such as GlobalGap, and the crucial role of supermarkets caused cooperatives to ally with other actors in various institutional arrangements [19, 33, 50]. The absence of environmental regulation was another critical factor preventing collective work [23]. Nowadays, cooperatives and other nonprofit organizations strive to affect or avoid pending or imminent regulations, such as eco-labels, certification schemes, management standards, and codes of conduct, by forming partnerships with private entities [51]. Likewise, the governments’ inefficiency in meeting sustainable developmental challenges and lack of support in developing countries causes cooperatives to realize their social responsibilities by interacting with other stakeholders in various partnerships [52].

In addition to external factors, developing the internal environment triggers a cooperative to address sustainability issues collectively. Leveraging resources was critical for partnerships to improve a cooperative’s productivity and efficiency [35]. All partners could benefit from social and financial capital to reduce the transaction costs and share the risks of amassing these resources independently [29]. When cooperatives and other actors participated in collection action, the acquired competencies (new knowledge and skills) motivated them [53]. Such expertise encouraged innovation capacity, essential in developing products and services, and, finally, promoted a cooperative’s competitiveness [54]. Finally, legitimacy-oriented motivations were among the factors in forming a partnership, and obtaining the resources for sustainability [23]. Gwiriri and Bennett [57] noted that legitimacy was crucial for cooperatives due to various aspects, such as becoming a more renowned actor, building a reputation, responding to accountability demands, and improving the sphere of impact. Finally, broader society-oriented motivations were highly critical for collaboration [55]. Such motivations originated from the pressing need for farmers and other partners to the strategic role of cooperatives in advocacy [56]. This role is discernible in different milieus, including promoting public awareness of issues, affecting policy regulations and legislations, driving environmental and social change, and addressing stakeholder problems [57].

### 2.3. Characterization of partnerships

Analyzing the characteristics supplied criteria employed to specify the nature of a partnership, compare various partnerships, and produce a plan for a joint discussion between organizations before entering into a partnership [58]. Kassem, Aljuaid [20] studied a partnership’s characteristics and underlined five essential areas: configuration, target people, objectives, stages, and typologies of partnerships. Analyzing the pattern of a partnership specified criteria used for partner selection [59]. Furthermore, it supplied information about partners’ nationalities and sectors [60]. The institutional form (Intra-sector or inter-sector), legal form, duration, and geographical coverage were the criteria helping analyze the configuration of a partnership [61]. To target people, agricultural cooperatives as business enterprises interacted with other actors to serve their members essentially and stretch their services to the society to conduct corporate social responsibility [62]. The beneficiaries served by the agricultural cooperatives depended on the specialization of a cooperative, the geographical focus, and the partnership’s objectives [14]. Beneficiaries might include a wide range of farmers in various fields, rural women, youth, the poor, or people with disabilities. Regarding the geographical coverage, a partnership between agricultural cooperatives and other partners was implemented at varying levels (village, city, governorate, region, national, or international), based on the partnership’s objectives and the available fund [59]. Formulating smart goals was critical for partnership sustainability [20]. The literature noted many purposes enacted by the cooperatives during collaborating, including access resources, capacity building, innovation and technology transfer, value chain development, agricultural market infrastructure development, and food security [63, 64].

Forming a successful partnership was not a straightforward process and was initiated through several stages, such as networking, coordination, cooperation, and collaboration [65]. In the initial networking step, partners exchange information for mutual benefit occurred. Limited time availability and trust characterized the relationship between partners in this stage. What was critical in this stage was exploring the “fit” between the partners [66]. The second included interchanging information and modifying activities for a common purpose, enriched by shifting toward greater coordination between the actors [38]. As the partnership developed into cooperation, tasks involved sharing resources, exchanging information, and modifying activities. It demanded a high level of trust between partners, a substantial amount of time, and partners’ ability to share resources. At this stage, there existed an increased interest in accountability and the initiation of thinking about the partnership’s governance [66]. The essential objective of the last phase (collaboration) is to improve the capacity of the other partner for mutual benefit and a common purpose. Pooling resources and sharing risks should be considered at this stage, and scaling was directed by a well-built governance system for the partnership [38].

Partnership typologies differed substantially across countries, organizations, and commodities. There existed no “one size fits all”–context matters [64]. The present study endorsed the classification of the partnership typologies depending on the business versus social orientation and the size of investments [67]. Per this classification, two classes of partnerships; that is, transactional and strategic partnerships. Transactional partnerships only included donating funds from the partners to cooperatives but not interacting further [58]. Transactional partnerships had two forms [68]; 1) commercial partnerships include exchanging payment, services, and goods for performing specific activities. The interaction of partners in such partnerships relied on contractual terms, including the size of sales and utilizing the partner’s products. 2) Philanthropic partnerships centering on charitable in-kind resources, time, or donation of funds. Partner interaction was restricted to accountability on fund distribution but could include little joint planning of priority areas. However, strategic partnerships produced a more crucial developmental effect by joining partners’ auxiliary strengths [58]. Thus, this type of partnership could be in two forms [69]: 1) partnerships forming independent value (new commercial initiatives). This type was the semi-strategic partnership, where all partners could achieve their individual goals jointly. The partnership produced value independently for both partners in varied ways. 2) Integrative partnerships: It was engineered strategically to tackle systematic issues and could bring substantial change across sectors and geographies. Nonetheless, partnerships stressing strategy over transaction had more relevance in acquiring the scale and depth of the effect needed to address complex issues.

### 2.4 Measuring success of partnerships

Measuring the success of partnerships was essential and should be constantly addressed in the monitoring and evaluation (M&E) plan [64]. It was crucial to understand how the activities, programs, or processes helped partners accomplish their objectives and business sustainability [58]. Such understanding manifested its potential in monitoring resources, accountability, reinforcing partners’ capacities to make informed decisions, adapt to unexpected circumstances, and drawing lessons [70]. Hence, the M&E system should measure the achieved outcomes, identify the responsibility of M&E tasks, and choose a suitable measurement method (source, tools, resources,…etc.) [71].

Outcomes of the partnerships differed according to organizational goals and involved one or more aspects from the following: resources (services, goods, technical and managerial expertise volunteers, and investments), corporate innovation; access to decision-makers; improved access to information; the development of human capital, and care for sustainability [23]. As per parties responsible for assessing the partnership, the responsibility lay in one partner or all engaged partners, or a third-party [59]. Lastly, the literature suggested various methodologies enacted by the partners to determine the success of partnerships. These methodologies could be different from each other as to the way and technique pursued to relate the partnership’s goals to the results achieved. One tool acknowledged in the literature about partnerships was the stakeholder satisfaction survey. It contained both open-ended and regular questions to judge the partnership’s outcomes from the stakeholder’s perspective [72]. Similarly, partnership members utilized a self-assessment tool to reveal their opinions and perceptions on varied aspects of the partnership experience [59]. Moreover, social return on investment (SROI) was used to examine the value of a partnership. SROI formed a holistic perspective and investigated a lucrative and valuable partnership. This view offered an opportunity to develop novel initiatives impacting social change for society [73]. However, a logic model was among the most crucial methodologies employed to assess the partnerships by various organizations. This methodology gauged the results chain (inputs, activities, outputs, and outcomes). It could underline the effect of a partnership in the short, intermediate, and long-term [74]. Ultimately, partnership evaluation literature, such as partnership effectiveness continuum [75], cost-benefit analysis [76], and social network analysis [77], utilized other methodologies.

**Fig 1:**
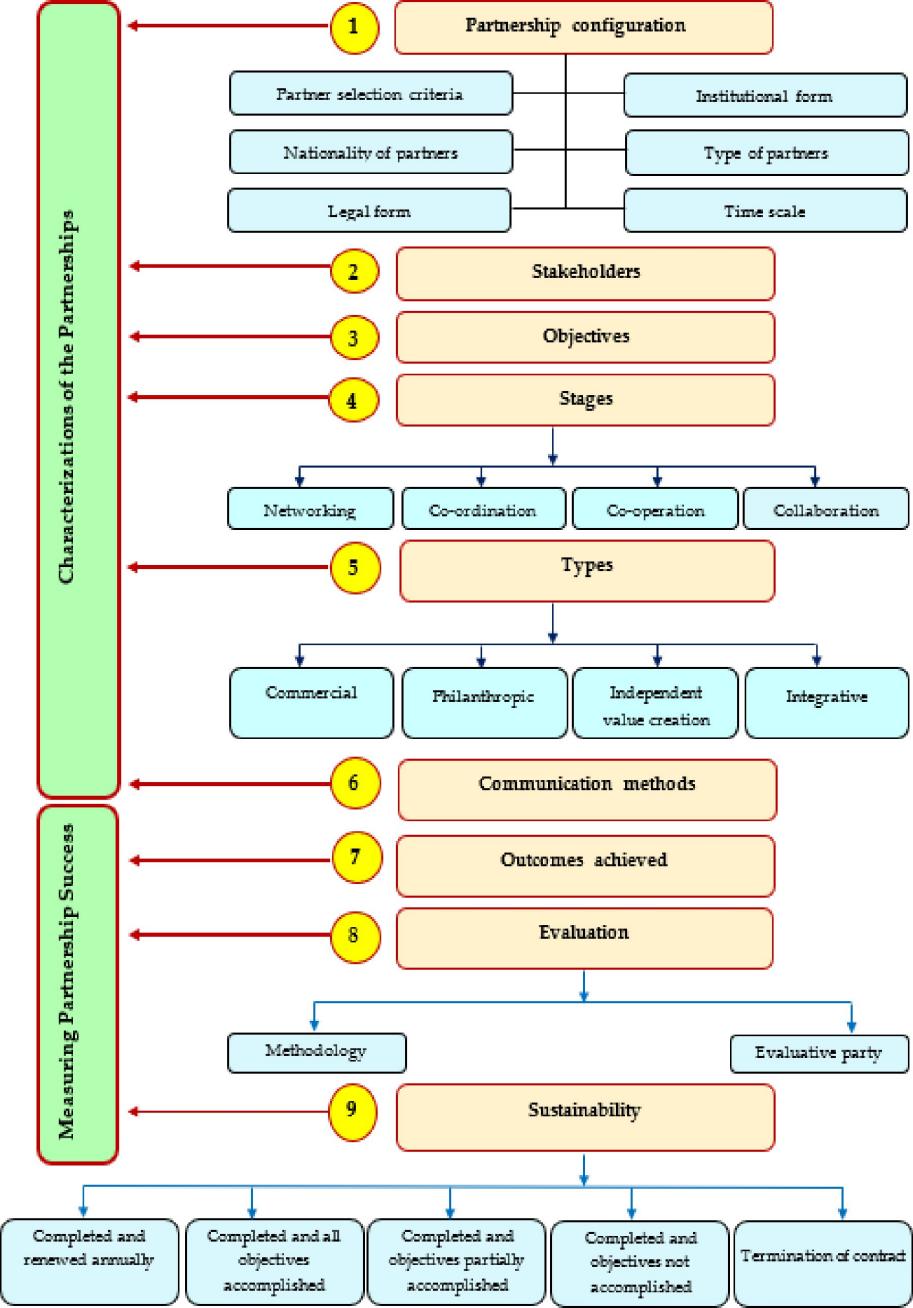
Analytical framework of the study.

Based on the literature review, the present study devised an analytical framework to achieve the objective (Fig 1), including two components: characteristics of the partnerships and partnership success. These components were grouped into nine sub-components: partnership configuration, stakeholders, objectives of the partnerships, partnership types, partnership stages, communication methods, achieved outcomes, partnership evaluation, and partnership sustainability. All sub-components and their indicators were assessed to examine the current partnerships between agricultural cooperatives and other actors in the study area.

## 3. Methodology

### 3.1 Ethics statement

Ethics approval was obtained from the Human Ethics Committee of King Saud University (Ref# HEC 2020/133). Verbally informed consent was obtained from all respondents involved in this study. All data collected are de-identified.

### 3.2 Research design and study area

The research strategy in the current study endorsed a qualitative research methodology utilizing a survey design. A phenomenological research type was performed to explain how the respondents experience a particular phenomenon (a partnership) from their own perspective [78]. The present study was conducted throughout Saudi Arabia (13 regions). Agricultural cooperatives were chosen as representative organizations. This methodology aimed to deliberate the results so that different actors would interact with agricultural cooperatives in partnerships in Saudi Arabia. The partnership was the unit of analysis in this study. Nonetheless, the selection of the partnerships was established by offsetting up inclusion and exclusion criteria. The inclusion criteria demanded that the cooperative sign the partnership between 2016 and 2020. Thus, informal partnerships were excluded. Furthermore, any partnership constructed and finalized before the period specified or built after this period was not considered the unit of analysis.

### 3.3. Participant selection

The present study population comprised all agricultural cooperatives functioning in Saudi Arabia (n = 66), as depicted in Table 1. Each cooperative was asked to gather data on partnerships with the other actors based on the specified inclusion criteria. Thus, all cooperatives included in the partnerships were chosen (n = 32). The number of the partnerships endorsed between 2016 and 2020 by these cooperatives was 69 (Table 1).

**Table 1:** Distribution of agricultural cooperatives participated in partnerships based on the regions of Saudi Arabia and the number of partnerships signed between 2016 and 2020.

Regions	Number of Agricultural Cooperatives	Number of Agricultural Cooperatives Participated in Partnerships	Number of Partnerships
Al-Riyadh	11	3	8
Al-Qaseem	7	4	11
Makkah	9	5	8
Al-Madinah	5	2	2
Hayel	5	2	7
Eastern	5	2	2
Al-Jouf	2	-	-
Northern Border	1	0	0
Tabouk	3	-	-
Aseer	7	5	13
Najran	1	-	-
Al-Baha	5	5	12
Jazan	5	4	6
Total	66	32	69

The (-) sign shows that no response has been received from some agricultural cooperatives.

### 3.4. Data collection and analysis

Depending on the analytical framework of this study (Fig 1), a semi-structured questionnaire was used in data collection. A previous study performed with nonprofit organizations was the basis for this study’s framework [20]. New sub-dimensions were added to the previous framework and some questions and selection of closed-ended questions determined the nature of agricultural cooperative work. The developed tool had two sections. Section one addressed queries on the partner selection criteria, partnership objectives, stakeholders, and communication methods. Concurrently, the rest of the variables relied on closed-ended questions. Assessing partnership success was included in section two. Open questions were used to specify the respondents’ responses about the methodology enacted in the partnership evaluation and outcomes obtained. The closed-ended questions gauged the other variables in this section. Each item in the tool was appraised and activated using previous studies in partnerships to guarantee content validity. Additionally, three expert academicians and three managers of agricultural cooperatives explored these items. The data collection methodology included face-to-face interviews with the managers of agricultural cooperatives. Moreover, content analysis was implemented for the partnership agreements and annexes and other official documents to acquire the required information depending on the analytical framework. This study took place between March and June 2021. Frequencies and percentages were utilized to present the results.

## 4. Results

### 4.1. Profile of participant agricultural cooperatives

The distribution of the agricultural cooperatives, based on the regions and number of the partnerships signed with other actors, is in Table 1. Of 13 areas in Saudi Arabia, the participant cooperatives that participated in the partnerships from 2016 to 2020 were studied in ten regions. Sixty-nine partnerships were finalized between the participant cooperatives (n = 32) and other actors, with the mean value of 2.15 partnerships for each cooperative. Aseer region had the highest number of partnerships (13) established by the cooperatives, followed by the Al-Baha region (12 partnerships).

Of 69 partnerships investigated (Table 2), most (57.9%) were signed between 2018 and 2019, while the least was in 2020 with 5.8%. The participant cooperatives attended divergent activities (Table 3). The results revealed that the partnerships signed changed across the main activities of participant cooperatives, where multi-purpose cooperatives had the highest number with a percentage of 46.8%. The results also suggested that the partnerships signed by multi-purpose cooperatives and bee-keeping partnerships accounted for about 60% of the total partnerships.

**Table 2:** Distribution of the partnerships signed between agricultural cooperatives and other actors based on the investigation period.

Year	Number*	%
2016	15	21.8
2017	10	14.5
2018	19	27.5
2019	21	30.4
2020	4	5.8
Total	69	100

* This number included the partnerships built before 2016 and still existed during the investigation.

**Table 3:** Distribution of partnerships and agricultural cooperatives according to the main activities of agricultural cooperatives.

Activity	Agricultural Cooperatives Participated in Partnerships			Partnerships Signed		
	Number		%	Number		%
Multi-purpose (Agriculture)	15		46.8	29		42.0
Bee-keeping	5		15.7	12		17.4
Poultry	1		3.1	3		4.3
Grain & animal feed	1		3.1	1		1.5
Fishery	2		6.3	3		4.3
Dates	2		6.3	3		4.3
Livestock	2		6.3	3		4.3
Marketing	1		3.1	6		8.7
Rose	1		3.1	3		4.3
Pomegranate	1		3.1	3		4.3
Olive	1		3.1	3		4.3
Total	32	100		69	100	

### 4.2. Characterizations of partnerships

#### 4.2.1 Configuration

**Fig 2:**
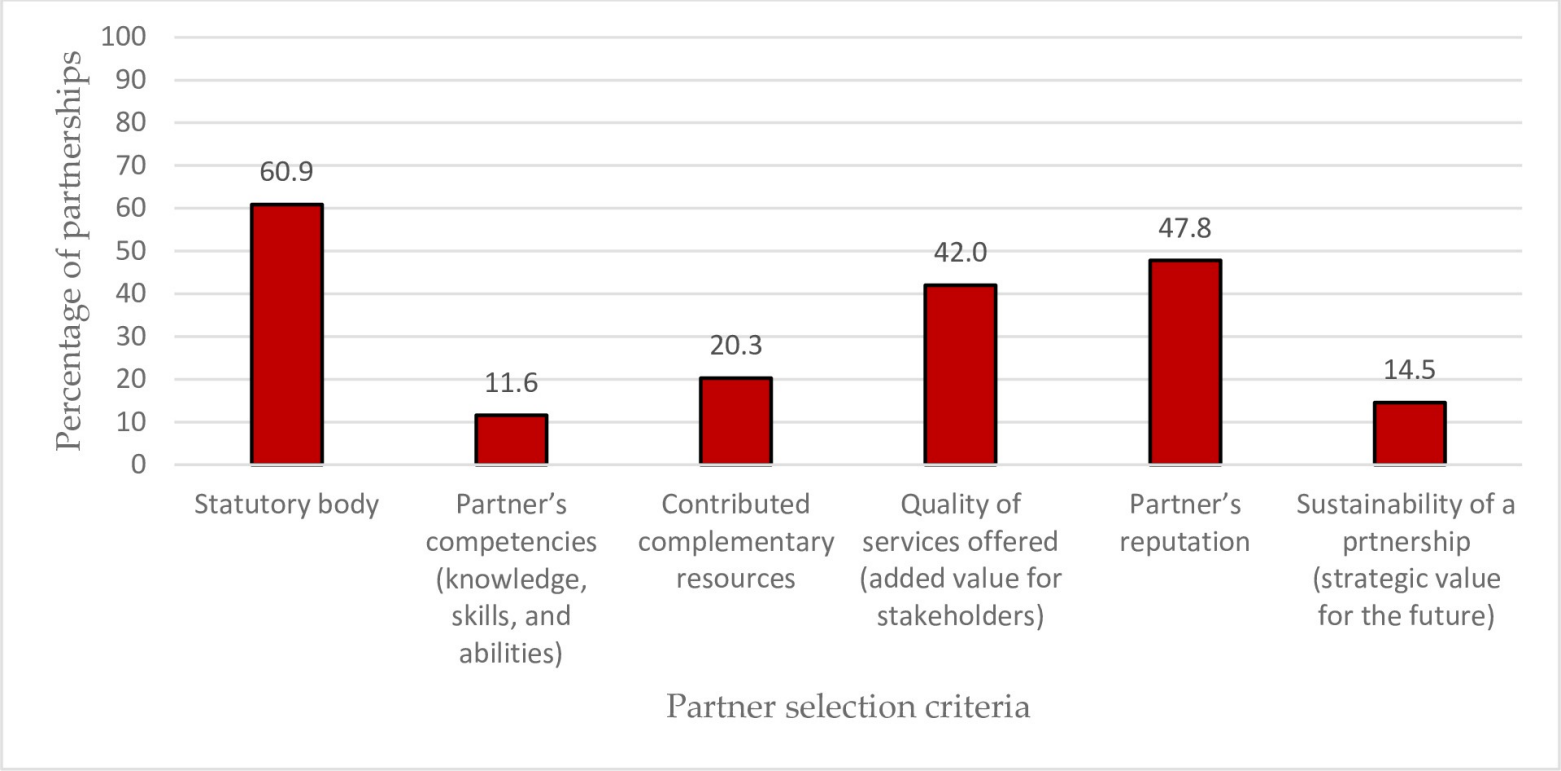
The partner selection criteria applied by the agricultural cooperatives.

The distribution of the partnerships as per the partner selection criteria is in Fig 2, where the agricultural cooperatives have noted various criteria for each. The results showed that statutory was the most crucial selection criterion (60.9%). The findings also underlined the relevance of the partner’s background as one of the essential selection criteria in less than half of the partnerships (47.8%). Moreover, the quality of services supplied by the partner ranked third with 42%.

**Fig 3:**
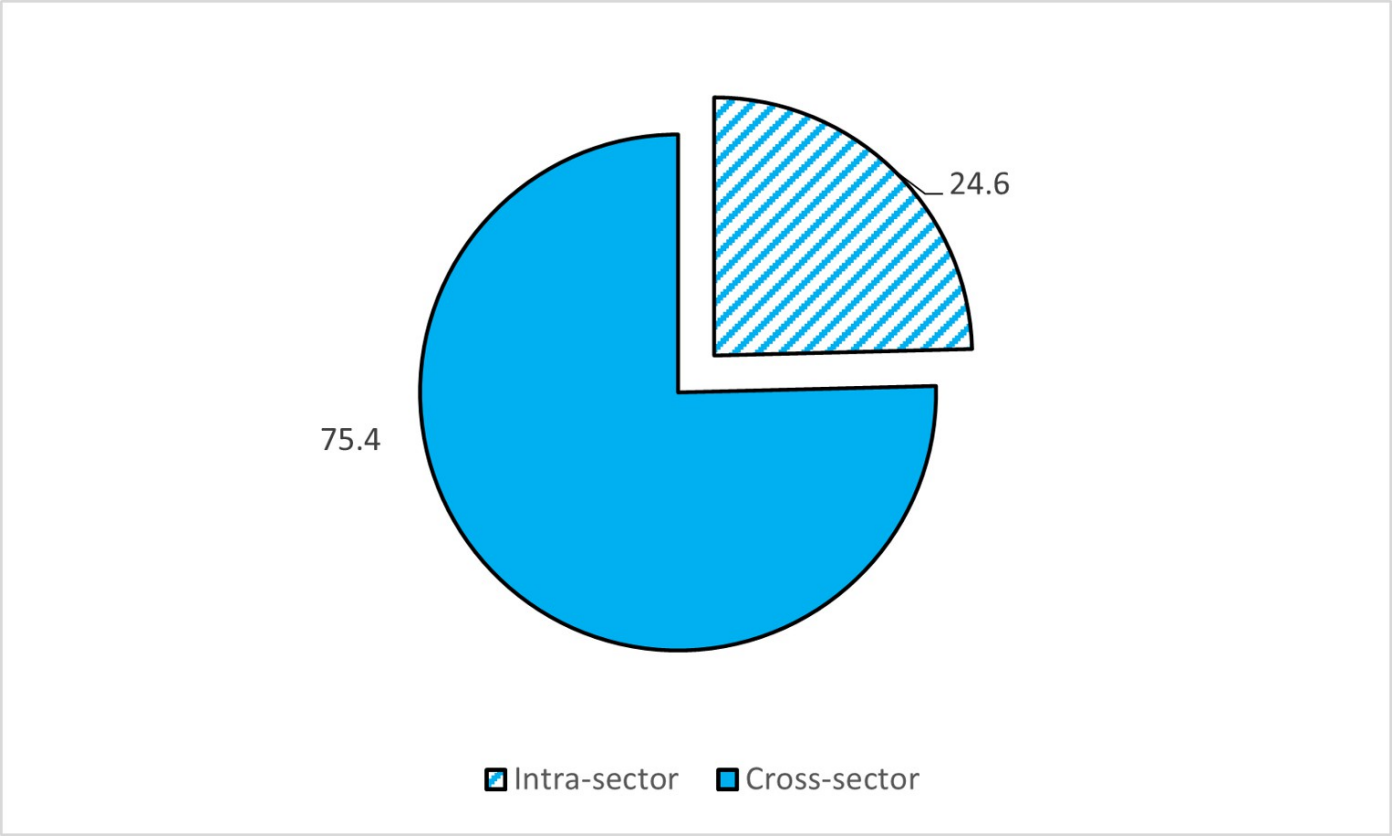
The institutional form of partnerships.

**Fig 4:**
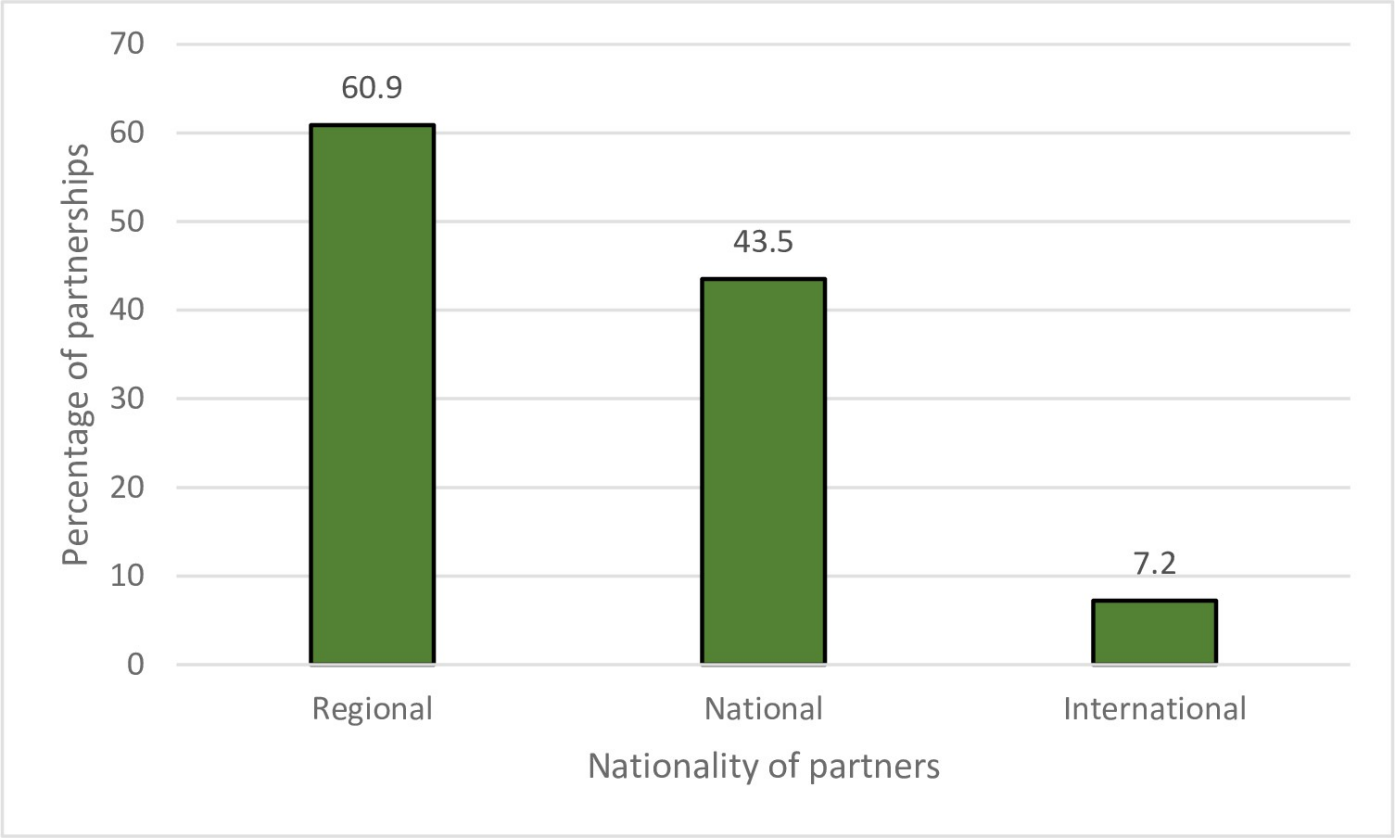
Nationality of actors participated in the partnerships.

**Fig 5:**
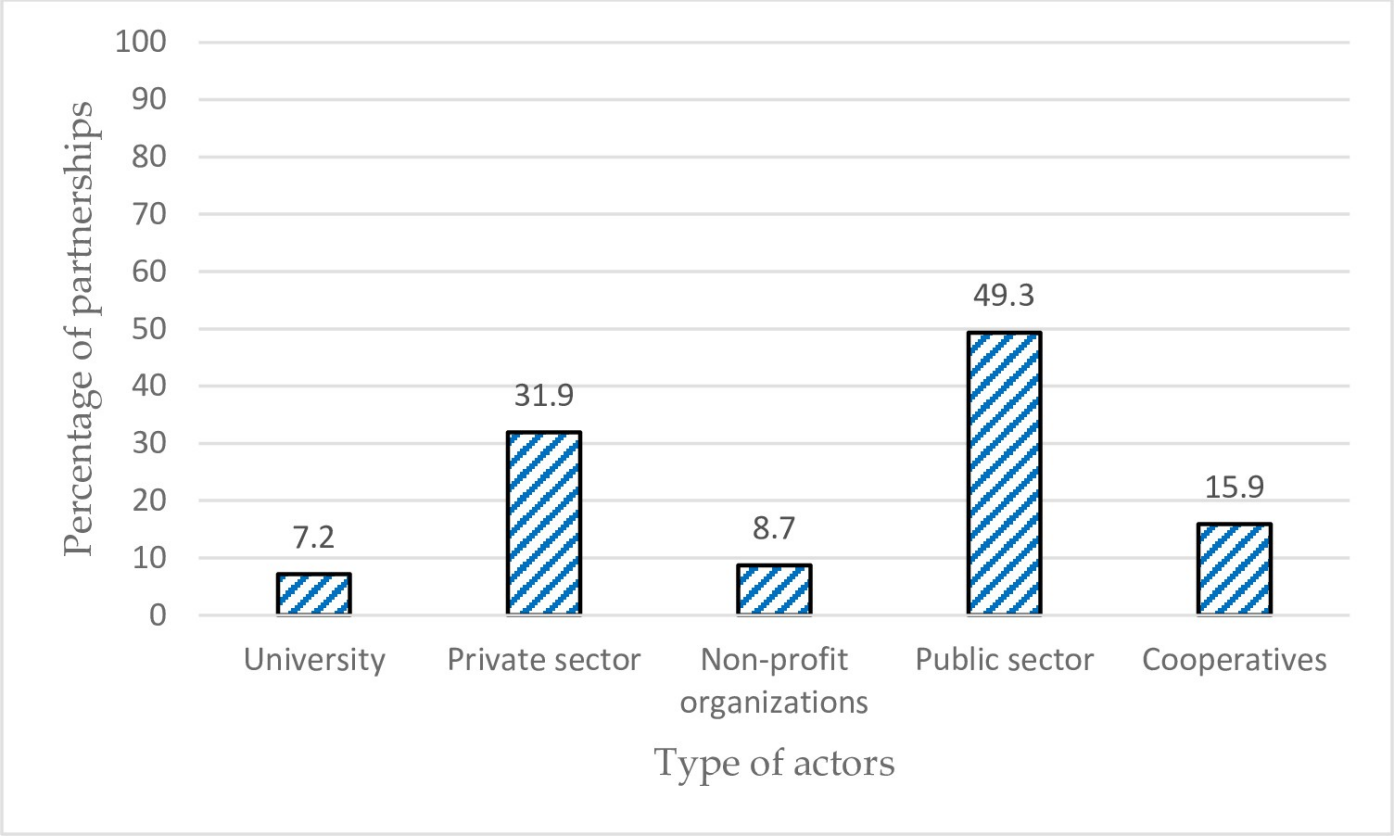
Type of actors participated in the partnerships (Some partnerships included more than one actor).

The institutional form of the partnerships is available in Fig 3. The findings revealed that more than three-quarters were cross-sectional partnerships. At the same time, the rest (24.6%) was in the social economy sector between agricultural cooperatives and other cooperatives or nonprofit organizations (intra-sector partnerships). The analysis revealed the variation in the nationality of partners (Fig 4). Nevertheless, the regional partners participated in the partnerships with 60.9%, followed by national partners (43.2%). About the actors participated, the findings in Fig 5 show that the types of actors are diverse and changes across the partnerships, covering the public sector (49.3%), private sector (31.9%), cooperatives (15.9%), nonprofit organizations (8.7%), and universities (7.2%).

**Fig 6:**
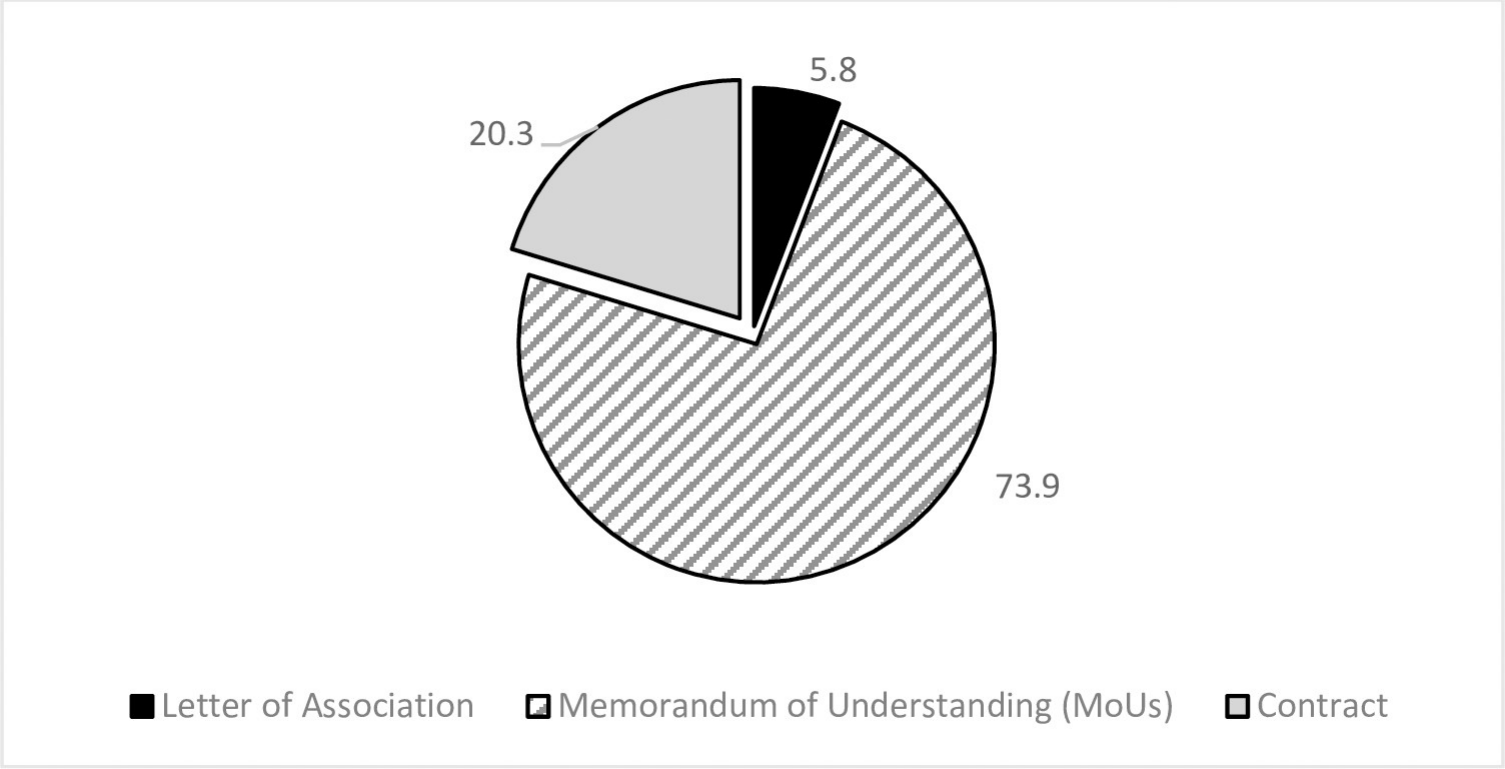
Distribution of the partnerships as per their legal form.

As part of the partnership’s configuration analysis, partnerships were investigated to specify the formal arrangements between partners, as shown in Fig 6. The results revealed that the memorandum of understanding was the most preferred legal arrangement in most of the partnerships (73.9%), suggesting that the parties favored non-binding written agreements to establish a partnership. On the contrary, the legally binding agreement (contract) explicitly specifying goals, roles, and responsibilities between partners occurred in 20.3%. Moreover, a written letter signed by the two parties (letter of association) occurred in 5.8% of the partnerships.

**Fig 7:**
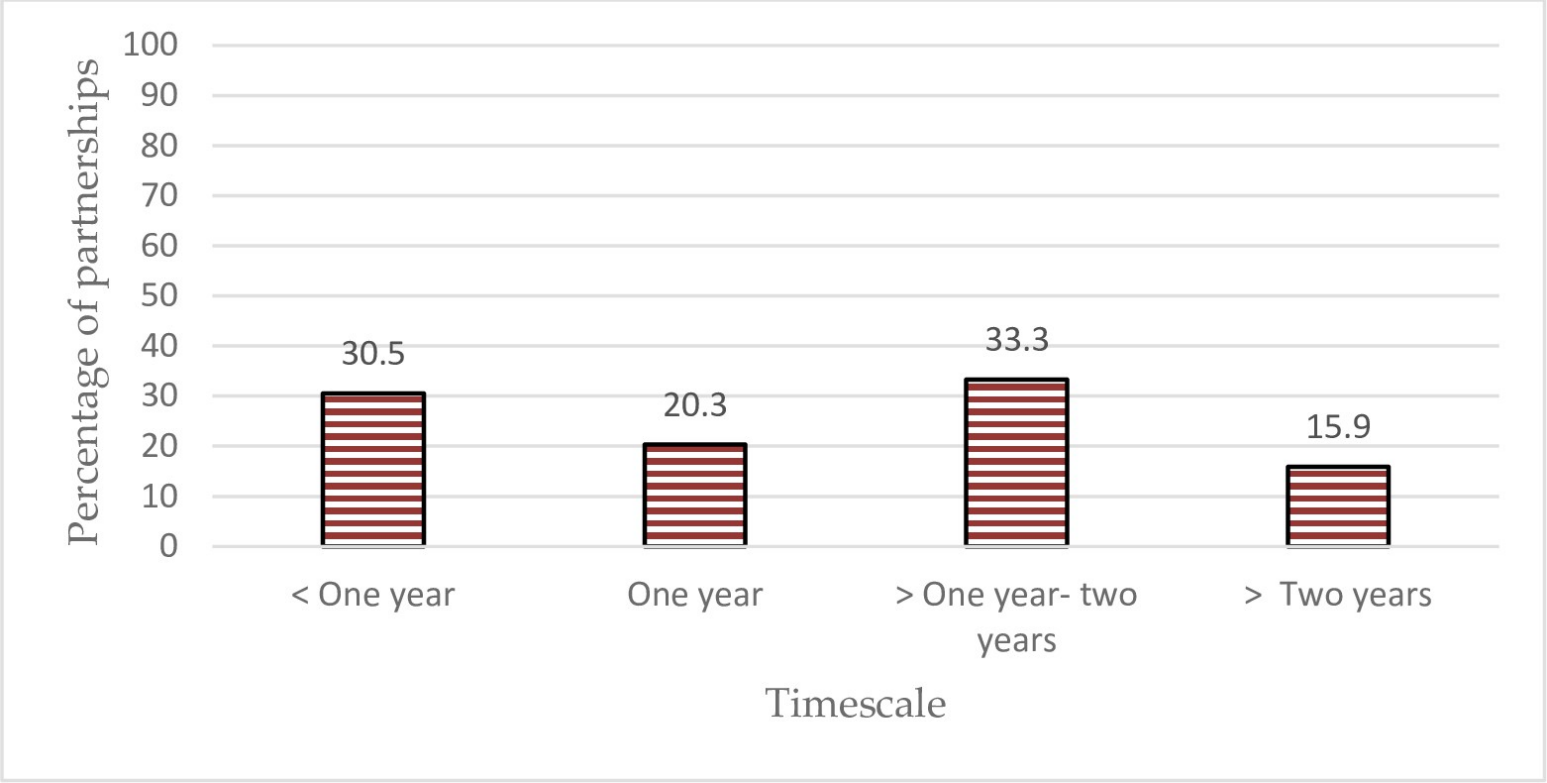
Distribution of the partnerships as per their timescale. * The duration of a partnership depicted has shown the term mentioned at the beginning of the collaboration. The extension periods of some are not available.

Fig 7 lists the duration of the partnerships. The results indicated that the timescale of the collaboration changed across the partnerships analyzed. About one third (33.3%) had the term ranging from a year to two years, followed by ones lasting less than a year (30.5%). 20.3% of partnerships lasted a year, while 15.9% persevered for more than two years.

**Fig 8:**
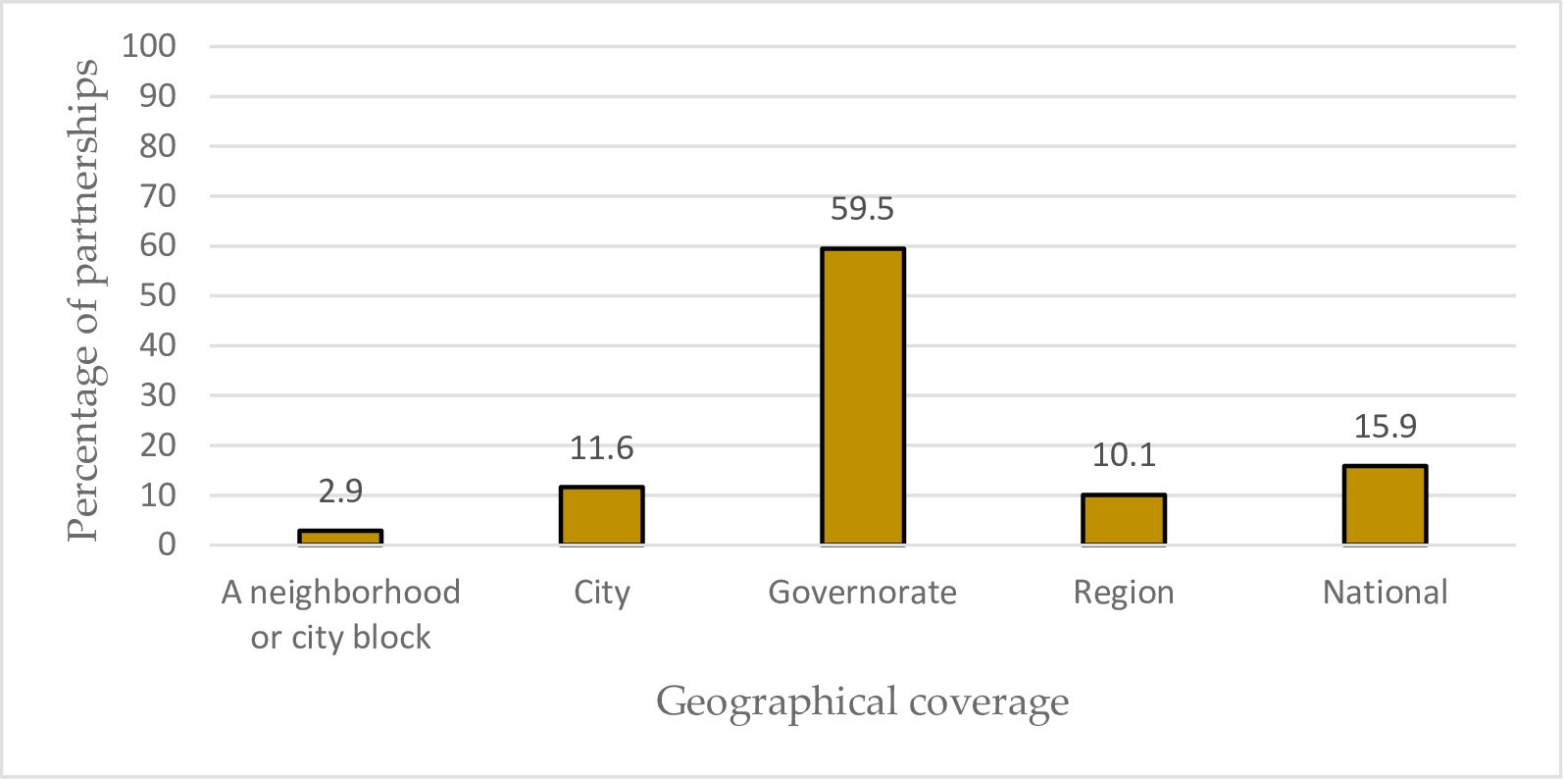
Distribution of the partnerships as per their geographical coverage.

As to the geographical coverage of the partnerships shown in Fig 8, most (59.5%) cover governorate-level areas. Furthermore, 15.9%had activities performed at the national level. Other geographical coverage areas included the city (11.6%), the region level (10.1%), and a specific neighborhood (2.9%).

#### 4.2.2 Stakeholders

Stakeholder types targeted by the partnerships are in Table 4, where some included various beneficiaries. Farmers and rural communities ranked first and second with 52.2% and 33.3%, respectively, while the beekeepers were third with about 22% of the partnerships. The livestock farmers, poultry farmers, fishers, university students, and the cooperative’s employees were other stakeholders targeted by the cooperatives during their interaction with other actors in the partnerships.

**Table 4:** Stakeholders of the partnerships.

Stakeholders	Frequency	%
Farmers	36	52.2
Beekeepers	15	21.7
Fishermen	3	4.3
Rural community	23	33.3
Livestock farmers	4	5.8
Poultry farmers	3	4.3
University students	1	1.4
Cooperative’s employee	1	1.4

#### 4.2.3 Objectives

Table 5 presents the objectives of the partnerships built between participant cooperatives and other actors. The results indicated that the most frequent goals for partnerships were agricultural services, training, capacity building, consulting and information support services, and event sponsorship. Additionally, providing marketing and advertising services (18.8%) and loans (15.9%) also called for collaboration. Lastly, other objectives covered educational and cultural services, indirect funding, direct funding, in-kind subsidies, recruitment, volunteering, entrepreneurship, and health services.

**Table 5:** Objectives of the partnerships.

Objectives	Frequency	%
Direct funding	5	7.2
Educational and cultural services	6	8.7
Administrative facilities	3	4.3
Recruitment	4	5.8
Volunteering	3	4.3
Indirect funding	6	8.7
Agricultural services (farming input supplies, equipment, etc.)	39	56.5
Training and capacity building	32	46.4
Marketing and advertising services	13	18.8
In-kind subsidies	4	5.8
Health services	1	1.4
Loans	11	15.9
Entrepreneurship	2	2.9
Consulting and information support services	28	40.6
Event organizing and sponsorship (workshops, festivals, etc.)	24	34.8

#### 4.2.4. Stages

The character of partnerships between partners is in Table 6, where it ranges on a continuum from networking to collaboration. The results uncovered that the highest number was in the second and third stages of interactive nature between partners with 37.7% and 36.2%, respectively. Thirteen partnerships (18.8%) centered on exchanging information between partners (Networking). However, the collaborative enriching of the capacity of the partners for a common purpose and mutual benefit, was slightly discernible in partnerships (7.3%).

**Table 6:** Stages of the partnerships.

Stages	Number	%
Networking	13	18.8
Coordination	26	37.7
Cooperation	25	36.2
Collaboration	5	7.3
Total	69	100

#### 4.2.5 Types

The distribution of partnership types about the degree of business versus social orientation is available in Table 7. The partnerships were distributed across the different types of transactional and strategic partnerships. Likewise, the results revealed that 44.9% of the partnerships could be labeled as transactional partnerships. These partnerships involved philanthropic (24.6%) and social investment (20.3%). Similarly, strategic partnerships showing the increased trend toward business for mutual benefit were discernible in 55.1% of the partnerships researched. New commercial partnerships were the most frequent strategic partnerships (44.9%), while 14.5% of the strategic partnerships could be labeled as core-business partnerships.

**Table 7:** Types of the partnerships.

Types	Number	%
A- Transactional partnerships		
Philanthropic	17	24.6
Social investments	14	20.3
B- Strategic partnerships		
New commercial initiatives	28	40.6
Core-business	10	14.5
Total	69	100

To underline the character of core business partnerships (ten partnerships), Table 8 shows the various types of services or business activities built by these partnerships. Even though multi-purpose cooperatives participated in 46.8% (Table 3), their effect in building core-business partnerships was scanty (20%). Most of the core-business partnerships molded by cooperatives were delineated in specific fields or activities. Private, public, and universities teamed up with cooperatives in 50%, 40%, and 10% of the core-business partnerships, respectively. A series of services or business activities were seen, including co-building factories, training centers, laboratories, quality systems, infrastructure projects in fish markets, and rehabilitation of the agricultural terraces.

**Table 8:** Distribution of the partnerships based on the field of services or business built, cooperatives’ activities, and partners participated.

Activity	Types of Partners	Field of Business/Service	Number	%
Agriculture (multi-purpose)	Public sector	• Rehabilitation of the agricultural terraces	1	20
	Private sector	• Coffee factory	1	
Bee-keeping	Private sector	• Training center • Bee quality laboratory	1	20
	Private sector	• Factory for manufacturing bee products	1	
Rose	University	• Research and technology transfer	1	10
Poultry	Private sector	• Factory for manufacturing poultry meat	1	20
	Public sector	• Development of the agricultural terraces	1	
Grain & animal feed	Public sector	• Grain and feed stores • Quality assurance system	1	10
Fishery	Private sector	• Ice factory	1	20
	Public sector	• Fish market infrastructure	1	
Total			10	

#### 4.2.6. Communication methods

The partnerships employed various communication methods to govern the relationship (Table 9). The results revealed that performing periodic meetings between partners was the most frequent, depicted in 46.4% of the total partnerships. Sending invitations for attending various events implemented within a partnership was the second with 34.8%. Nonetheless, written communications and social media share were 29% and 21.8%, respectively.

**Table 9:** Communication methods followed in the partnerships.

Methods	Frequency	%
Meetings	32	46.4
Social media	15	21.8
Written communications (e-mail, letters, reports, etc.)	20	29.0
Invitation for attending various events	24	34.8

### 4.3 Measuring success of the partnerships

#### 4.3.1 Outcomes

After completing a partnership for both the associations and the partners, the outcomes acquired are in Table 10. The findings revealed that solving agricultural problems for society was the priority (43.8%) from the cooperatives’ perspective. However, organizational innovation, human capital development, and adequate services supply were essential benefits in more than a third of the partnerships. From the partners’ perspective, polishing the partner’s image in media was the most beneficial, with 46.4% of the partnerships. Moreover, noticing the increased recognition of the partner’s role in social responsibility and advertising for the partner’s services was the most crucial outcome with 37.7% and 34.8% of the partnerships, respectively.

**Table 10:** Outcomes for both cooperatives and partners.

Outcomes	Frequency	%
A- Cooperatives		
Gaining expertise from the partners	14	20.3
Increased access to financial capital	13	18.8
Human capital development	22	31.9
Enhanced reputation	16	23.2
Organizational innovation	26	37.7
Solving agricultural problems for society	30	43.8
More effective services	21	30.4
B- Partners*		
Polishing the partner’s image in media	32	46.4
Advertising for the partner’s services	24	34.8
Reducing tax rates deducted from the partners	4	5.8
Increased recognition of the partner’s role in social responsibility	26	37.7
Mutual participation in decision making	11	15.9
Honoring the partner’s contribution in events	5	7.2
Increased number of clients for partners	2	2.9
Benefiting from cooperative’s expertise	19	27.5
Offering discounts on cooperative’s services to partners	1	1.4

* The benefits for the partners were determined from the cooperatives’ perspective.

#### 4.3.2 Evaluation

Table 11 presents that only 17.2% of the partnerships have been evaluated by following frameworks or tools. Of these, 36.4% were assessed by the cooperatives or the partnerships individually, while the third party appraised 27.2%. A limited number of assessment methodologies occurred across the partnerships. The stakeholder satisfaction survey was employed to assess 54.5% of the total. Likewise, the other partnerships used the self-assessment tool, social return on investment, and logic model.

**Table 11:** Types of methodologies used for the partnership assessment.

Variable	Number	%
Use of a methodology to assess the partnerships * (n = 64)		
Yes	11	17.2
No	53	82.8
Assessing party ** (n = 11)		
Cooperative	4	36.4
Partner	4	36.4
Third-party	3	27.2
Methodologies used for the partnership assessment ** (n = 11)		
Stakeholder satisfaction survey	6	54.5
Partnership self-assessment tool	2	18.2
Logic Model	2	18.2
Social return on investment	1	9.1

* Five partnerships were still implemented during data collection

** Number of assessed partnerships

#### 4.3.3 Continuum of the partnership’s sustainability

To determine the success of partnerships, the situation after completing the partnerships between participant cooperatives and other actors is in Table 12. Surprisingly, more than a third analyzed (37.5%) were annually renewed, suggesting the sustainability of a partnership. Nevertheless, 31.3% attained their objectives within the timescale of a partnership. However, the percentage not wholly achieving the planned goals was 28.1%. A 3.1% of contracts was terminated due to not getting partners’ confidence.

**Table 12:** Sustainability continuum of the partnerships.

Variable	Number	%
Completed and renewed annually	24	37.5
Completed and all objectives accomplished	20	31.3
Completed and objectives partially accomplished	18	28.1
Completed and objectives not accomplished	-	-
Termination of contract	2	3.1

* Five partnerships were still implemented during data collection.

## 5. Discussion

In the current study, the objective was to analyze the characteristics of partnerships signed between agricultural cooperatives and development actors. This objective should clarify the complete picture of how various actors strive to join in the agricultural cooperatives for sustainability, what institutional structures participate in, the extent of success in achieving the planned objectives, and where policy gaps exist. Accordingly, this study systematically examined 69 partnerships molded between agricultural cooperatives and other actors from 2016 to 2020 at the national level in Saudi Arabia. The findings obtained from this study would support the third theme of the national transformation plan (2021–2025) of the 2030 vision (promote social development and enhance social economy). It also would help its strategic objectives: to support the growth of social economy sector and strengthen the social economy organizations to attain more profound impact [79].

The study findings suggested that less than half of the total agricultural cooperatives in Saudi Arabia participate in partnerships with other actors. Likewise, a small number of partnerships were built during the investigation period (five years). These results revealed that establishing partnerships was demanding for cooperatives due to varied obstacles. Such obstacles included the knowledge and expertise needed of cooperatives in attracting other actors for collaboration, molding and governing partnerships, marketing services and capabilities, or other organizational, and legislative issues hindering the establishment of partnerships. Thus, the interviews with the managers of cooperatives should help provide some notes and some facts about the cooperative sector in Saudi Arabia. The number of agricultural cooperatives in Saudi Arabia cannot meet the real needs of the agricultural community, population density, or international standards due to the small number of cooperatives and their members. The reason for the hesitation in building cooperative societies is twofold [80]: First, the commercial activities between 2000 and 2015 were open, and the owners of the companies worked individually when their activities grew, as they did not require cooperation. Second, business people did not acknowledge the principle of cooperative work because they only concentrated on their institutions and companies.

Although governments worldwide upheld and promoted the involvement of the private sector to achieve SDGs, interactions of cooperatives with the private sector were not evident in the majority of partnerships. However, the private sector had acute effects on sustainable development, including economic growth, job creation, and the provision of goods and services [81]. The changes in private sector’s traditional role with other actors could generate critical added value with the growing global challenges, the complexity of issues, increased interest in sustainability, and demands to stimulate corporate social responsibility strategies [82]. Put differently, pursuing the principle that the whole is greater than the sum of its parts, divergent interests might change into new sources of innovation, and partners carry the potential to solve their problems jointly [83]. Therefore, the governments devised and enacted frameworks to guarantee good governance of partnerships between cooperatives and the private sector and to subdue the organizational and legislative barriers for partnering. Thus, Manning and Roessler [84] noted that the private sector involvement in partnerships with cooperatives could be mitigated by the brokers (third parties) performing a critical bridging role to balance partners’ interests. Stadtler and Probst [85] stressed that brokers could serve three functions: a convener, a mediator, or a learning catalyst during the life cycle of a partnership. These roles allow brokers to help partners devise sustainability-oriented partnerships.

The results revealed that more than half of the partnerships’ orientation toward business partnerships was evident. This finding illustrated that 37.5% were renewed after completion. Nonetheless, most of these partnerships concentrated on developing individual value rather than co-working in devising service or solving a specific problem for mutual benefit. It could be due to a lack of direct public support; thus, cooperatives should find new financing models to achieve their mission in the long term. This orientation enables cooperatives to acquire expertise, construct their capacity, leverage resources, and utilize a partnership to their competitive advantage [23]. Shifting from philanthropic to strategic partnerships needs analyzing the partner’s motivation to specify the “sweet spot” between the business opportunities and development goals [58]. Furthermore, attracting partners by planning marketing-oriented activities adds to the businesses’ public image [83]. Understanding such issues allow cooperatives to gain from strategic partnerships as sustainable finance for their actions [63].

The present study does not address performing a systematic assessment to determine the effects of the partnerships examined in the short and long run. Most partnerships relied on assessing stakeholders’ or partners’ views on partnership processes and outcomes of the assessment methodologies employed. Little evidence was observed for measuring the effects and exploring the results chain by endorsing tools such as the logic model and SROI. The lack of sufficient funds may explain these results for contracting with third-party; the lack of knowledge about the relevance of assessment and how partners could benefit from them in devising future partnerships. They may originate from the lack of employee’s skills to plan and implement evaluation methodologies professionally. These results were in line with the study of Kassem, Aljuaid [20], performed in Saudi Arabia on nonprofit organizations. They discovered that only 20.3% of the partnerships built between nonprofit organizations and other actors were assessed. However, the absence of assessment methodologies does not enable organizations and individuals to evaluate the progress of a partnership, uncover mistakes, achieve experience and knowledge, present a solid basis for transparency and accountability [74, 75]. Thus, producing systematized evidence on the efficiency of the partnerships is debatable [83]. Therefore, partners should understand that the monitoring and evaluation component during the planning and development is critical in a partnership’s life-cycle [61, 72]. Partners also should reach a consensus about the outcomes of the partnership’s activities, agree on how the achievement of outcomes will be measured and evaluated, and specify the programs and actions needed to achieve the outcomes [86].

## 6. Conclusions

This study explores the partnerships built between agricultural cooperatives and other actors in Saudi Arabia. Although the number of partnerships signed between 2016 and 2020 is not many, more than half are strategic. Moreover, most partnerships are renewed after completion or finalized after attaining the planned objectives. Solving stakeholders’ agricultural programs and organization innovation are the most critical outcomes of the partnerships for agricultural cooperatives. The public sector is the principal actor who participated in agricultural cooperatives in the partnerships among all actors. Additionally, conducting a systematic assessment of partnerships has not received adequate attention from the partners. The analysis of the partnerships’ characteristics in this study have implications in both theory and practice. This paper offers an anlytical framework for anlyzing the characteristics of partnerships. The developed analytical framework conceptualizes the interactions between the agricultural cooperatives and development actors and offers a practical guide to assist future researchers who want to analyze the characteristics of partnerships.

Practically, this paper provides insights into the gaps that needs to be filled by the agricultural cooperatives in developing and managing partnerships. One of the notes derived from agricultural cooperatives’ participation relates to increasing their collaborations with the private sector, specifically in the core business partnerships guaranteeing sustainability. Therefore, cooperatives should develop ways to promote partnerships, including preparing market analysis and economic feasibility studies for their proposals, analyzing stakeholders’ needs, understanding partners’ motivations for partnering, and constant capacity building for their employees. Thus, brokers need to intervene between partners to encourage practices and augment the partners’ roles during the entire life cycle. Furthermore, the study suggests developing marketing approaches for agricultural cooperatives utilizing social media platforms. This strategy is critical to improving a cooperative’s visibility and partners’ image, publicizing the results, and sharing lessons learned with other cooperatives. Enhancing assessment practices is a critical issue and needs tackling. Co-developing performance indicators and improving cooperative employees’ skills in assessment methodologies are crucial in monitoring and evaluation. The evaluation of a partnership should cover the following three essential aspects: the actual costs and benefits of the partnership approach, the added value for the partners, and effects on the stakeholders and society. From a policy perspective, as small number of cooperatives participate in partnerships, policymakers should use this situation to design and manage a multi-stakeholder platform to encourage the dialogue between cooperatives and diverse actors. Such platforms can help build trust across the actors and enrich understanding of their alignment of interest and the benefits of partnering. The platforms can affect systematically. There are some limitations needing acknowledgment. The present study focuses on gathering data on partnerships from the cooperatives’ perspective, without considering the other partners’ perspectives. Moreover, the governance structure of a partnership is not part of the analytical framework. Future studies should include these aspects. These studies may improve our understanding of the value of partnerships from different perspectives and investigate how the governance structure upholds a partnership’s processes.
